# National clinical reaudit on managing adults with bullous pemphigoid 2024 highlighting shifting clinical practices

**DOI:** 10.1093/skinhd/vzaf063

**Published:** 2025-11-06

**Authors:** Ravi Ramessur, Hayley Smith, Zoe C Venables, Tanya Tumbeva, M Firouz Mohd Mustapa, David A R de Berker, the British Association of Dermatologists’ Clinical Standards Unit, D A R de Berker, R Ramessur, H Smith, Z C Venables, C Charman, A Shaw, S Seddik, T Tumbeva, M F Mohd Mustapa

**Affiliations:** St John’s Institute of Dermatology, School of Basic & Medical Biosciences, Faculty of Life Sciences & Medicine, King’s College London, London, UK; Watford General Hospital, West Hertfordshire NHS Trust, Watford, UK; Department of Dermatology, Sheffield Teaching Hospitals NHS Foundation Trust, Sheffield, UK; Department of Dermatology, Norfolk and Norwich University Hospitals Foundation Trust, Norwich, UK; Norwich Medical School, University of East Anglia, Norwich, UK; Clinical Standards Unit, British Association of Dermatologists, London, UK; Clinical Standards Unit, British Association of Dermatologists, London, UK; Department of Dermatology, Bristol Royal Infirmary, Bristol, UK

## Abstract

**Background:**

Bullous pemphigoid (BP) is a chronic, autoimmune, blistering disorder that predominantly affects older adults and is associated with significant morbidity and treatment challenges. The British Association of Dermatologists (BAD) clinical guideline for managing people with BP was published in 2012; a national clinical audit was undertaken in 2018. This 2024 reaudit evaluates changes in clinical practice, including diagnostic methods, treatment strategies and documentation standards.

**Objectives:**

To reassess compliance with BAD audit standards, compare findings with the 2018 audit and identify trends in BP management.

**Methods:**

Over 9 weeks in 2024, BAD members submitted data for 450 cases of BP from 77 centres across the UK. Audit standards included documentation of comorbidities, osteoporosis risk management, patient satisfaction and systemic treatment monitoring.

**Results:**

The reaudit identified a shift in diagnostic practices, an increased proportion of severe baseline disease and continued gaps in ­osteoporosis risk documentation. The use of doxycycline as a primary treatment has increased significantly since 2018.

**Conclusions:**

The findings highlight both progress and persistent challenges in BP management. Improved documentation and greater adherence to osteoporosis management guidelines remain priorities for future practice.

## Teaser

Bullous pemphigoid (BP) is the most common autoimmune blistering disorder affecting older adults, and this 2024 re-audit aimed to evaluate changes in diagnostic methods, treatment strategies and guideline adherence compared with the 2018 audit. Data from 450 BP cases across 77 UK centres revealed a shift towards indirect immunofluorescence for diagnosis, an increased use of doxycycline as first-line therapy, and a higher proportion of severe disease at presentation. The findings highlight ongoing challenges in osteoporosis risk management, variability in practice and a need for updated guidelines to better reflect evolving clinical practice and the growing role of teledermatology.

What is already known about this topic?

- Bullous pemphigoid (BP) is the most common autoimmune blistering disease in Western populations, primarily affecting older adults.
- Management guidelines emphasize corticosteroids, immunomodulatory agents and doxycycline for treatment, alongside careful monitoring of comorbidities and osteoporosis risk.
- The 2018 national clinical audit identified gaps in documentation of comorbidities and osteoporosis management, while systemic treatment and patient satisfaction documentation showed stronger adherence.

What does this study add?

- The 2024 reaudit highlights an increased prevalence of severe BP at presentation, potentially reflecting delays in care.
- A significant shift toward indirect immunofluorescence for diagnosis was observed, while documentation for both osteoporosis risk and bone protection therapy declined.
- Use of doxycycline increased markedly, yet oral corticosteroids remain widely used.
- Persistent gaps in guideline adherence emphasize the need for standardized documentation and targeted education.

Bullous pemphigoid (BP) is the most common autoimmune blistering disorder in Western populations.^1^ A recent study in the UK from 1998 to 2017 identified incidence to be 7.6 per 100 000 person-years with incidence increasing with age, in particular in older men.^2^ Characterized histologically by subepidermal blisters and on immunofluorescence by autoantibodies against hemidesmosomal antigens, BP typically manifests as pruritus alongside tense bullae on an erythematous or urticarial base.^3^ The condition significantly impacts on patients’ quality of life, particularly in severe cases, and is associated with a range of comorbidities, notably neurological and psychiatric conditions.^4^ BP also carries a risk of mortality, mainly due to sepsis, particularly in older adults with underlying systemic diseases.^5,6^

Traditionally, management of BP has centred on the use of oral corticosteroids, which remain effective but pose risks of adverse effects such as hyperglycaemia, hypertension, osteoporosis and infections.^7^ The 2012 British Association of Dermatologists (BAD) clinical guideline for BP recommended treatment with high-potency topical corticosteroids as the first-line treatment for localized disease, and oral corticosteroids for more extensive involvement.^8^ Disease-modifying antirheumatic drugs (DMARDs) such as azathioprine, methotrexate and mycophenolate mofetil are included as treatment options for steroid-sparing purposes. Anti-inflammatory antibiotic therapies, particularly doxycycline, were introduced into routine clinical practice later, as safer alternatives based upon evidence from the 2017 Bullous Pemphigoid Steroids and Tetracyclines (BLISTER) trial, which demonstrated that doxycycline was noninferior to oral prednisolone for short-term blister control for BP and significantly safer in the long term.^9^

The BAD guideline emphasized the importance of comprehensive management, incorporating regular monitoring of systemic treatment, documentation of comorbidities and prevention of osteoporosis in oral corticosteroid-treated patients. These were formalized into four audit standards to facilitate their implementation and assessment. The first BP national clinical audit in 2018 revealed variability in compliance with documentation standards particularly across regions.^10^ While patient satisfaction and systemic treatment monitoring showed higher adherence, there was a lower frequency of documentation of comorbidities and osteoporosis risk management.

The 2024 reaudit provides an opportunity to evaluate progress in BP management over the past 5 years, particularly in the context of evolving diagnostic and therapeutic approaches. Additionally, the audit reflects the potential impact of wider healthcare challenges, such as the COVID-19 pandemic, on the care of patients with BP. By evaluating compliance with the original audit standards and identifying areas for improvement, this study aims to inform future clinical practice and guideline updates.

## Materials and methods

The 2024 reaudit adhered to the standards established in the 2012 BAD clinical guidelines,^8^ and employed the same methodological framework as the 2018 audit.^10^ BAD members were invited to participate via email, with reminders sent weekly during the 9-week data collection period (12  February–15 April 2024). Each participating centre was asked to submit data for five consecutive adults with BP who had been under dermatology supervision (in part or completely) for at least 12 months.

Data collection focused on the following areas:

- Diagnostic methods, including direct and indirect immunofluorescence and clinical diagnosis.
- Disease severity was classified according to the number of blisters at presentation: very mild (<3 blisters), mild (3–10 blisters), moderate (11–30 blisters) and severe (>30 blisters).
- Documentation of comorbidities, specifically diabetes and hypertension.
- Osteoporosis risk assessment and management in corticosteroid-treated patients.
- Patient satisfaction with treatment outcomes.
- Use and monitoring of systemic treatments, including baseline and follow-up testing.

An anonymized, standardized Microsoft Excel (Version 16.88, © 2024 Microsoft) proforma was used to collect and collate data. Statistical comparisons with the 2018 audit were performed using χ^2^ tests using R (version 4.1.2; R Foundation for Statistical Computing, Vienna, Austria), with statistical significance set at *P* < 0.05.

## Results

### Participation and patient demographics

**Figure 1:**
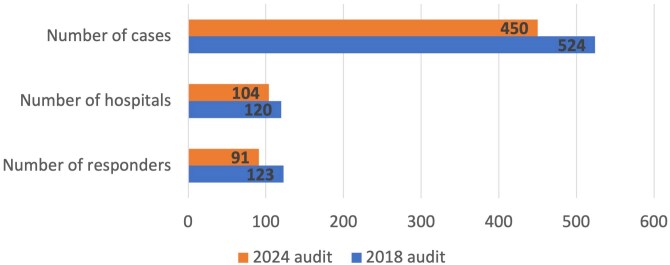
Bar chart showing the number of responders, hospitals and cases.

**Figure 2:**
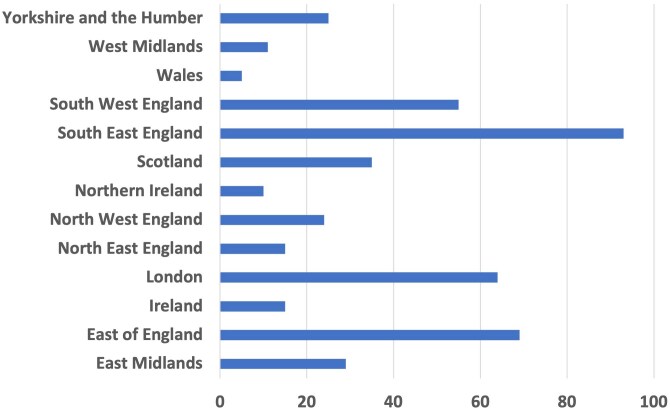
Bar chart showing the number of patients included in the audit across regions of the UK and Ireland.

**Figure 3:**
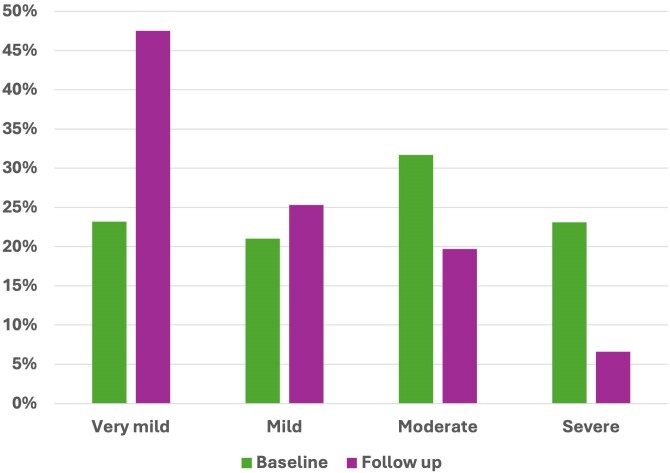
Bar chart showing the baseline severity of patient population compared with severity at second follow-up.

Ninety-one responders from 77 centres submitted data for 450 cases, representing a response rate of 32.2% (calculated based on the number of centres responding), which was higher than the 24.7% recorded in 2018 (Figure 1). South East England was the region that contributed the highest number of cases (Figure 2). The median age of patients was 78 years (interquartile range 70–84). Disease severity at baseline was recorded for 324 cases (72%), with a notable increase in severe presentations (>30 blisters) vs. 2018 (23.1% vs 5.4%, *P* < 0.001) (Figure 3). Cases of very mild disease (<3 blisters) were significantly less common in 2024 (23.2%) compared with 2018 (63.9%, *P* < 0.001).

### Diagnostic practices

**Figure 4:**
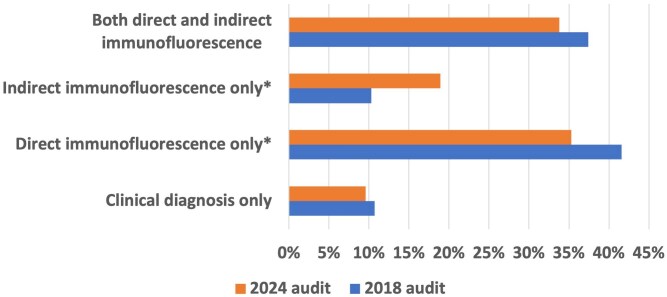
Bar chart demonstrating the percentage of patients with bullous pemphigoid diagnosed clinically and those diagnosed with immunofluorescence. *P < 0.05.

The proportion of cases diagnosed using direct immunofluorescence decreased from 41.6% in 2018 to 35.3% in 2024 (*P* = 0.04), while the use of indirect immunofluorescence increased significantly (10.3% in 2018 vs. 18.9% in 2024, *P* = 0.03) (Figure 4). Diagnoses made solely on clinical grounds were similar, at approximately 10%.

### Compliance with audit standards

#### Documentation of comorbidities

**Figure 5:**
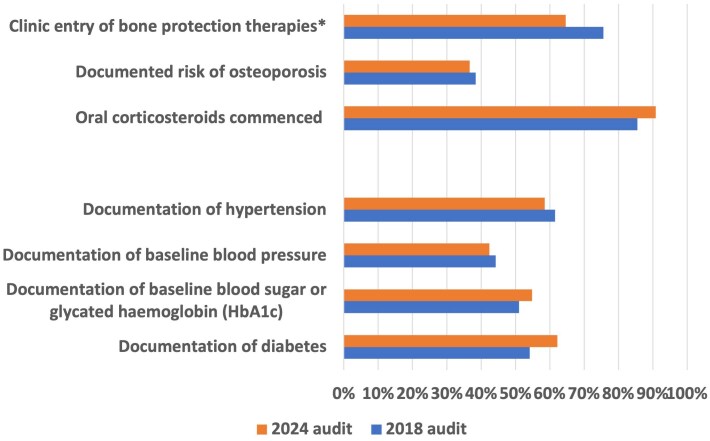
Bar chart demonstrating the percentage for ‘yes’ responses for bullous pemphigoid audit standards. *P < 0.05.

Records of diabetes history were available for 62.2% of patients, while hypertension documentation was recorded in 58.5% (Figure 5). These rates showed minimal improvement compared with those in 2018 (54.1% and 61.5%, respectively).

#### Osteoporosis risk management

Osteoporosis risk assessment in patients on oral steroids was documented in 36.7% of cases, similar to the finding in 2018 (38.4%) (Figure 5). However, a decline in the proportion of patients prescribed bone protection therapy if on oral steroids was observed, from 75.6% in 2018 to 64.6% in 2024 (*P* = 0.004).

#### Patient satisfaction

**Figure 6:**
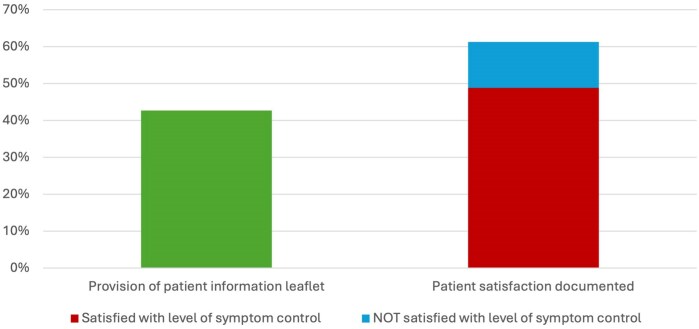
Bar chart showing the percentage of ‘yes’ responses to having recorded documentation of patient satisfaction, that the patient was satisfied with the level of symptom control and provision of a patient information leaflet.

Documentation of patient satisfaction increased from 59.3% of cases in 2018 to 65.1% in 2024 (Figure 6). Among these, 85.6% of patients expressed satisfaction with their symptom control.

#### Systemic treatment practices

**Figure 7:**
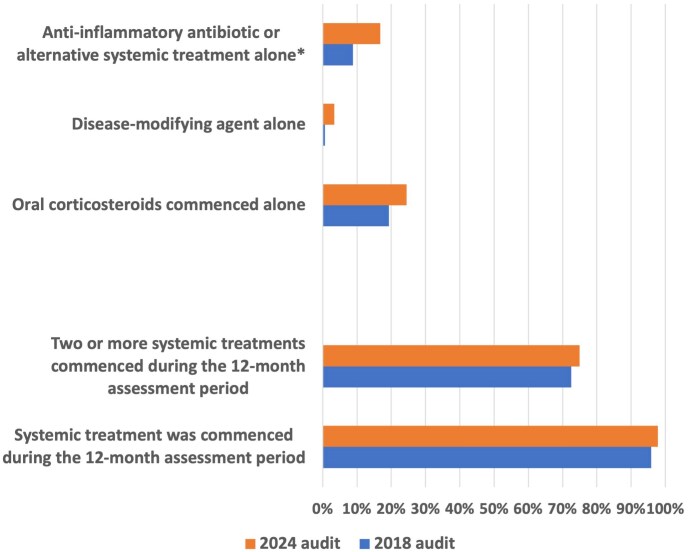
Bar chart demonstrating systemic treatments used in bullous pemphigoid (BP). *P < 0.05.

Oral corticosteroids were prescribed in 90.9% of cases, compared with 85.5% in 2018 (Figure 7; Table 1). An increase in the use of doxycycline was observed, with 83.8% of patients receiving it in 2024 vs. 50.7% in 2018 (*P* < 0.001). Mycophenolate mofetil replaced azathioprine as the most commonly used DMARD (13.3% vs. 8.4%). Use of methotrexate also increased, compared with the 2018 audit (from 3.1% in 2018 to 8.0% in 2024).

**Table 1:** All recorded treatments to treat bullous pemphigoid: 2018 audit data vs. 2024 audit data

Category	Medications	Percentage of patients receiving specific treatment	
		2018 audit	2024 audit
Topicals (creams, ointments, etc.)	Clobetasol propionate	66.8	74.9
	Betamethasone valerate	7	4.4
	Mometasone furoate	7	7.1
	Fusidic acid + betamethasone	3.6	2.9
	Clotrimazole + betamethasone	1.0	0.4
	Clobetasone butyrate	0	3.8
	Hydrocortisone	0	1.8
Oral/systemic steroids	Prednisolone	84.9	90.9
Antibiotics	Doxycycline	50.7	83.8
	Lymecycline	7.6	6.4
	Minocycline	3.4	1.1
	Erythromycin	0.2	1.8
	Oxytetracycline	1.0	0.4
Immunosuppressants	Methotrexate	3.1	8.0
	Azathioprine	11.2	8.4
	Mycophenolate	11.1	13.3
	Intravenous immunoglobulin	0	0.2
	Rituximab	0	0.7
Vitamins	Nicotinamide	4.0	11.8
	Niacinamide	2.6	1.1

## Discussion

Audit is essential to assess current practice, ensure guideline adherence and inform future guideline changes. The findings of the 2024 reaudit demonstrate not only progress in terms of changing practice reflecting new clinical evidence and technologies, but also ongoing challenges regarding the impact of, and recovery from, the COVID-19 pandemic.

A significant shift in diagnostic practices was observed, with reduced reliance on direct immunofluorescence (41.6% in 2018 vs. 35.3% in 2024) and increased use of indirect methods either alone (10.3% in 2018% vs. 18.9% in 2024) or in conjunction with direct immunofluorescence (37.4% in 2018 vs. 33.8% in 2024). While this change may address logistical challenges of performing a biopsy in older patients, it is also likely reflective of the increasing use of teledermatology following the COVID-19 pandemic. Many of these patients, who are often frail and older, can be effectively managed remotely, especially if they are already in residential or nursing home care. Although indirect immunofluorescence may provide a feasible diagnostic alternative in such scenarios, it raises concerns about diagnostic accuracy, as direct immunofluorescence remains the gold standard.^11^ Clinicians may need further guidance on balancing the shift towards remote care while ensuring diagnostic rigour.

The increasing burden of the older population on healthcare services compounds this issue. Over the next 20 years, the UK population aged 85 years and over is projected to increase from 1.6 million to 2.6 million.^12^ This demographic shift underscores the importance of adapting healthcare delivery models, including teledermatology, to address the specific needs of an ageing population.

The increased severity of disease at baseline compared with that in 2018 may reflect delays in care, resulting in more severe disease at the point of care, or may reflect increasing use of teledermatology which means that mild cases are being managed more in the community. These delays may have been exacerbated by the COVID-19 pandemic, which disrupted routine healthcare access and potentially contributed to later-stage presentations in secondary care.

A notable decline in documentation for osteoporosis risk management was observed, with apparently fewer patients receiving bone protection therapy despite high level of corticosteroid use; this could be due to poorer documentation compared with cases in 2018. This decline could also suggest gaps in guideline adherence and highlights the need for targeted educational initiatives. Enhanced documentation tools, such as standardized clinic templates, checklists or proformas, may help address this issue and improve compliance.

The rise in doxycycline use reflects the growing influence of evidence-based practice following the BLISTER trial.^9^ The safety profile for doxycycline and its ­noninferiority to prednisolone for short-term blister control make it an increasingly attractive option, particularly for older patients with comorbidities. However, the continued high reliance on oral corticosteroids suggests that clinicians remain cautious about transitioning fully to alternative therapeutic options.

The shift away from azathioprine as a steroid-sparing drug towards mycophenolate mofetil was also observed. This trend reflects mycophenolate’s more favourable toxicity profile and efficacy.^13^ The recognition of causal links between azathioprine and cutaneous squamous cell carcinoma^14^ may have also influenced clinicians’ preference for alternative agents with lower, long-term risk profiles. These changes highlight the importance of ongoing research to refine treatment strategies for BP. Dapsone was not recorded as a treatment choice in this audit. While it is used for BP in some countries, UK prescribing patterns favour corticosteroids, doxycycline and immunosuppressants. This may reflect differences in clinician preference, local guidelines and patient comorbidities.

Despite emerging evidence supporting the use of biologics and small molecules in BP,^15^ their uptake remains limited in routine UK practice. In this audit, rituximab was recorded in only 0.7% of cases, while no other biologic or ­small-molecule agents were documented. This may, in part, reflect restricted National Health Service funding and consequent access for these therapies in the context of BP. As further evidence accumulates and access improves, future audits may capture an increased role for these agents in BP management.

This study has several limitations, including potential selection bias, as the identification and inclusion of cases were determined by the participating centres, despite guidance from the study group to ensure consecutive case selection. Inpatients with more severe disease are likely to have been identified more readily than those with milder cases of BP managed through teledermatology, which might account for the increase in the proportion of patients with more severe BP identified. Another limitation is the lack of data on whether patients were managed as inpatients or outpatients at initial diagnosis, which may have influenced treatment decisions.

Additionally, participation bias may be present, as centres with a greater interest or expertise in BP management may have been more likely to participate. Retrospective data collection may underestimate compliance due to incomplete records, and variability in regional practices further complicates direct comparisons. Higher response rates and clearer guidance on case inclusion would improve the generalizability of findings within and across regions.

The 2024 reaudit highlights areas of significant changes, namely to clinical presentation of and practice for managing BP, including increased disease severity at presentation and shifting diagnostic and treatment practices. While improvements in patient satisfaction documentation are encouraging, persistent gaps in osteoporosis management and variability in practice remain areas to be addressed through greater awareness and better evidence.

The shift towards teledermatology in clinical practice and indirect diagnostic methods emphasizes the need for a balance between resource constraints and diagnostic accuracy, especially as the older population continues to grow. Furthermore, the observed shifts in therapeutic choices, including increasing reliance on doxycycline and mycophenolate mofetil, underline the need for updated guidelines to reflect these changes. Existing guidelines, with the BAD’s now being over 12 years old, may no longer reflect current evidence and practice. Consideration should also be given to the development of a ‘living’ guideline to ensure that future recommendations can adapt dynamically to emerging and evolving evidence, practices and therapeutic developments.

## Data Availability

The data underlying this article will be shared on reasonable request to the corresponding author.

## Appendix 1

This is a national clinical audit report prepared for the British Association of Dermatologists (BAD) Clinical Standards Unit, which includes the National Audit Sub-Committee. Members of the BAD’s Clinical Standards Unit who have been involved are D A R. de Berker (Chair, National Audit Sub-Committee), R Ramessur, H Smith, Z C Venables, C Charman, A Shaw, S Seddik, T Tumbeva (Clinical Standards Project Coordinator) and M F Mohd Mustapa (Director of Clinical Standards).

## References

[1] Langan SM, Smeeth L, Hubbard R et al Bullous pemphigoid and pemphigus vulgaris–incidence and mortality in the UK: population based cohort study. BMJ 2008; 337:a180. 18614511 10.1136/bmj.a180 PMC2483869

[2] Persson MSM, Harman KE, Vinogradova Y et al Incidence, prevalence and mortality of bullous pemphigoid in England 1998–2017: a population-based cohort study. Br J Dermatol 2021; 184:68–77. 32147814 10.1111/bjd.19022

[3] Bernard P, Antonicelli F. Bullous pemphigoid: a review of its diagnosis, associations and treatment. Am J Clin Dermatol 2017; 18:513–28. 28247089 10.1007/s40257-017-0264-2

[4] Sánchez-García V, Pérez-Alcaraz L, Belinchón-Romero I, Ramos-Rincón JM. Comorbidities in patients with autoimmune bullous disorders: hospital-based registry study. Life (Basel) 2022; 12:595. 35455086 10.3390/life12040595 PMC9031095

[5] Joly P, Benichou J, Lok C et al Prediction of survival for patients with bullous pemphigoid: a prospective study. Arch Dermatol 2005; 141:691–8. 15967914 10.1001/archderm.141.6.691

[6] Roujeau J-C, Lok C, Bastuji-Garin S et al High risk of death in elderly patients with extensive bullous pemphigoid. Arch Dermatol 1998; 134:465–9. 9554299 10.1001/archderm.134.4.465

[7] Cortés B, Khelifa E, Clivaz L et al Mortality rate in bullous pemphigoid: a retrospective monocentric cohort study. Dermatology 2012; 225:320–5. 23257934 10.1159/000345625

[8] Venning VA, Taghipour K, Mohd Mustapa MF et al British Association of Dermatologists’ guidelines for the management of bullous pemphigoid 2012. Br J Dermatol 2012; 167:1200–14. 23121204 10.1111/bjd.12072

[9] Williams HC, Wojnarowska F, Kirtschig G et al Doxycycline versus prednisolone as an initial treatment strategy for bullous pemphigoid: a pragmatic, non-inferiority, randomised controlled trial. Lancet 2017; 389:1630–8. 28279484 10.1016/S0140-6736(17)30560-3 PMC5400809

[10] Smith H, Mohd Mustapa MF, Cheung ST, de Berker DAR. National audit on the management of bullous pemphigoid. Clin Exp Dermatol 2020; 45:289–94. 31502666 10.1111/ced.14086

[11] Oh H, Kim CH, Lee YJ. Bullous pemphigoid diagnosis: the role of routine formalin-fixed paraffin-embedded skin tissue immunochemistry. Sci Rep 2022; 12:10519. 35732698 10.1038/s41598-022-14950-z PMC9217790

[12] Office for National Statistics. National population projections: 2020-based interim 2024. Available at: https://www.ons.gov.uk/peoplepopulationandcommunity/populationandmigration/populationprojections/bulletins/nationalpopulationprojections/2020basedinterim (last accessed 14 January 2025).

[13] Beissert S, Werfel T, Frieling U et al A comparison of oral methylprednisolone plus azathioprine or mycophenolate mofetil for the treatment of bullous pemphigoid. Arch Dermatol 2007; 143:1536–42. 18087004 10.1001/archderm.143.12.1536

[14] Inman GJ, Wang J, Nagano A et al The genomic landscape of cutaneous SCC reveals drivers and a novel azathioprine associated mutational signature. Nat Commun 2018; 9:3667. 30202019 10.1038/s41467-018-06027-1 PMC6131170

[15] D’Agostino GM, Rizzetto G, Marani A et al Bullous pemphygoid and novel therapeutic approaches. Biomedicines 2022; 10:2844. 36359364 10.3390/biomedicines10112844 PMC9687138

